# Keratinocytes use FPR2 to detect *Staphylococcus aureus* and initiate antimicrobial skin defense

**DOI:** 10.3389/fimmu.2023.1188555

**Published:** 2023-05-31

**Authors:** Marco Lebtig, Jasmin Scheurer, Marie Muenkel, Janna Becker, Effie Bastounis, Andreas Peschel, Dorothee Kretschmer

**Affiliations:** ^1^ Department first: Infection Biology, Interfaculty Institute of Microbiology and Infection Medicine, University of Tübingen, Tübingen, Germany; ^2^ Cluster of Excellence EXC 2124 Controlling Microbes to Fight Infections, University of Tübingen, Tübingen, Germany; ^3^ Department of Dermatology, University of Tübingen, Tübingen, Germany; ^4^ Interfaculty Institute of Microbiology and Infection Medicine, University of Tübingen, Tübingen, Germany

**Keywords:** keratinocytes, skin colonization, formyl-peptide receptor 2, *Staphylococcus aureus*, inflammation

## Abstract

**Introduction:**

Keratinocytes form a multilayer barrier that protects the skin from invaders or injuries. The barrier function of keratinocytes is in part mediated by the production of inflammatory modulators that promote immune responses and wound healing. Skin commensals and pathogens such as *Staphylococcus aureus* secrete high amounts of phenol-soluble modulin (PSM) peptides, agonists of formyl-peptide receptor 2 (FPR2). FPR2 is crucial for the recruitment of neutrophils to the sites of infection, and it can influence inflammation. FPR1 and FPR2 are also expressed by keratinocytes but the consequences of FPR activation in skin cells have remained unknown.

**Methods:**

Since an inflammatory environment influences *S. aureus* colonization, e. g. in patients with atopic dermatitis (AD), we hypothesized that interference with FPRs may alter keratinocyte-induced inflammation, proliferation, and bacterial colonization of the skin. To assess this hypothesis, we investigated the effects of FPR activation and inhibition in keratinocytes with respect to chemokine and cytokine release as well as proliferation and skin wound gap closure.

**Results:**

We observed that FPR activation induces the release of IL-8, IL-1α and promotes keratinocyte proliferation in a FPR-dependent manner. To elucidate the consequence of FPR modulation on skin colonization, we used an AD-simulating *S. aureus* skin colonization mouse model using wild-type (WT) or Fpr2^-/-^ mice and demonstrate that inflammation enhances the eradication of *S. aureus* from the skin in a FPR2-dependent way. Consistently, inhibition of FPR2 in the mouse model or in human keratinocytes as well as human skin explants promoted *S. aureus* colonization.

**Discussion:**

Our data indicate that FPR2 ligands promote inflammation and keratinocyte proliferation in a FPR2-dependent manner, which is necessary for eliminating *S. aureus* during skin colonization.

## 1 Introduction

The skin acts as a physical and immunological protective barrier with over 90% keratinocytes as the predominant cell type in the epidermis. Keratinocytes respond to pathogenic microorganisms and injury by producing antimicrobial peptides and cytokines (1). During their maturation from the basal to the uppermost layer, epidermal cells undergo a calcium-regulated differentiation. Staphylococci are ubiquitous colonizers of human skin, especially coagulase-negative staphylococci as *S. epidermidis*. A less frequent colonizer of the skin represents the opportunistic pathogen *Staphylococcus aureus* (2)*. *S. aureus** causes a wide range of diseases from superficial skin infections to severe invasive infections such as septicemia or endocarditis (3). Acute bacterial skin infections are a common reason to seek healthcare facilities, and *S. aureus* is the most common organism associated with hospital-acquired infections (4). These infections are further complicated by methicillin-resistant *S. aureus* (MRSA) strains, which are prevalent in hospitals. Virulence of pathogenic *S. aureus* depends on various virulence factors, e. g. the phenol-soluble modulin (PSM) peptide toxins. *S. aureus* can not only induce cutaneous infections but it also frequently contributes to flare-up of inflammatory skin diseases such as atopic dermatitis (AD) (5). AD is characterized by acute eczematous, pruritic lesions over dry skin and severely impairs the quality of life of those affected. Under normal circumstances *S. aureus* rarely colonizes human skin. In AD, *S. aureus* frequently colonizes unaffected skin. Interestingly, it has been shown that an inflammatory environment promotes *S. aureus* skin colonization (6, 7).

Formyl-peptide receptors (FPRs) belong to the family of chemoattractant G-protein coupled receptors (GPCRs), which are critical for detecting bacterial infections and are known to influence inflammation (8). All bacteria release short formylated peptides, ligands of FPR1. However, staphylococci, especially highly pathogenic *S. aureus* strains, additionally secrete large amounts of PSMs, which represent FPR2 ligands (9). Neutrophils express FPR1 as well as FPR2 and FPR activation is known to transiently increase intracellular calcium, chemotaxis, degranulation, expression of receptors for phagocytosis and thereby enhance pathogen elimination (8, 10). Although it has been shown that various epithelial cells including keratinocytes also express FPR1 and FPR2 (11), the consequences of FPR activation in these cells are poorly understood. FPR2 ligands such as the synthetic ligand WKYMVM and the human antimicrobial peptide LL-37 were shown to increase keratinocyte proliferation and improve wound healing (12, 13). In contrast, FPR inhibitors attenuated phorbol 12-myristate 13-acetate (PMA)-induced ear edema by reducing local production of cytokines such as MCP-1, CXCL1 as well as IL-6, and thereby possess anti-inflammatory properties (14). Opposite effects were reported in intestinal epithelial cells, whose stimulation with the formylated peptide fMLF, an FPR1 agonist, decreased TNFα-induced NFκB signaling and proinflammatory cytokine production (15). Most AD patients are colonized by *S. aureus* and experience relapses of their skin disorder because of overgrowth of this bacterium (16). Studies revealed that topical administration of anti-inflammatory corticosteroids or tacrolimus lowered *S. aureus* levels in atopic skin (17, 18). *S. aureus* releases high amounts of FPR2 ligands, the PSMs. PSMs are short formylated α-helical, amphipathic peptides and are released in high amounts especially by pathogenic *S. aureus* strains, e. g. USA300 (19). Therefore, we hypothesized that keratinocyte-dependent FPR2 activation induces skin inflammation.

Since it is unclear if and how FPRs influence bacteria-induced skin inflammation such as AD, we investigated the consequences of FPR activation or inhibition in the presence or absence of *S. aureus* colonization in human keratinocytes, and in an animal model of skin inflammation and *S. aureus* colonization using wild-type (WT) and Fpr2-/- mice. We observed that the FPR1 ligand fMLF and the FPR2 ligands PSMα3, PSMα, PSMε and MMK1 induced IL-8 as well as IL-1α and in part MIP-3α, and CXCL10 release by human keratinocytes. The FPR inhibitors tBOC and WRW4 prevented the FPR ligand-induced release of IL-1α and IL-8 as well as proliferation of keratinocytes. In addition, we found enhanced *S. aureus* colonization but not *S. epidermidis* colonization in FPR2 inhibitor-treated keratinocytes and in human skin explants. Accordingly, we observed that FPR2 inhibition in an *in vivo* model simulating human AD enhanced *S. aureus* colonization only in WT but not in Fpr2^-/-^ mice. This enhanced *S. aureus* colonization correlated with a decreased release of inflammatory cytokines IL-1α, IL-1β as well as MIP-2 in WT but not in FPR2^-/-^ mice. Our data show that induction of skin inflammation by *S. aureus* is mediated at least in part by FPR2 activation in keratinocytes leading to enhanced IL-8 as well as IL-1α release and keratinocytes proliferation. Conversely, inhibition of FPR2 in keratinocyte prevents inflammation but promotes *S. aureus* skin colonization.

## 2 Results

### 2.1 Expression of FPRs by keratinocytes and activation by bacterial ligands

**Figure 1:**
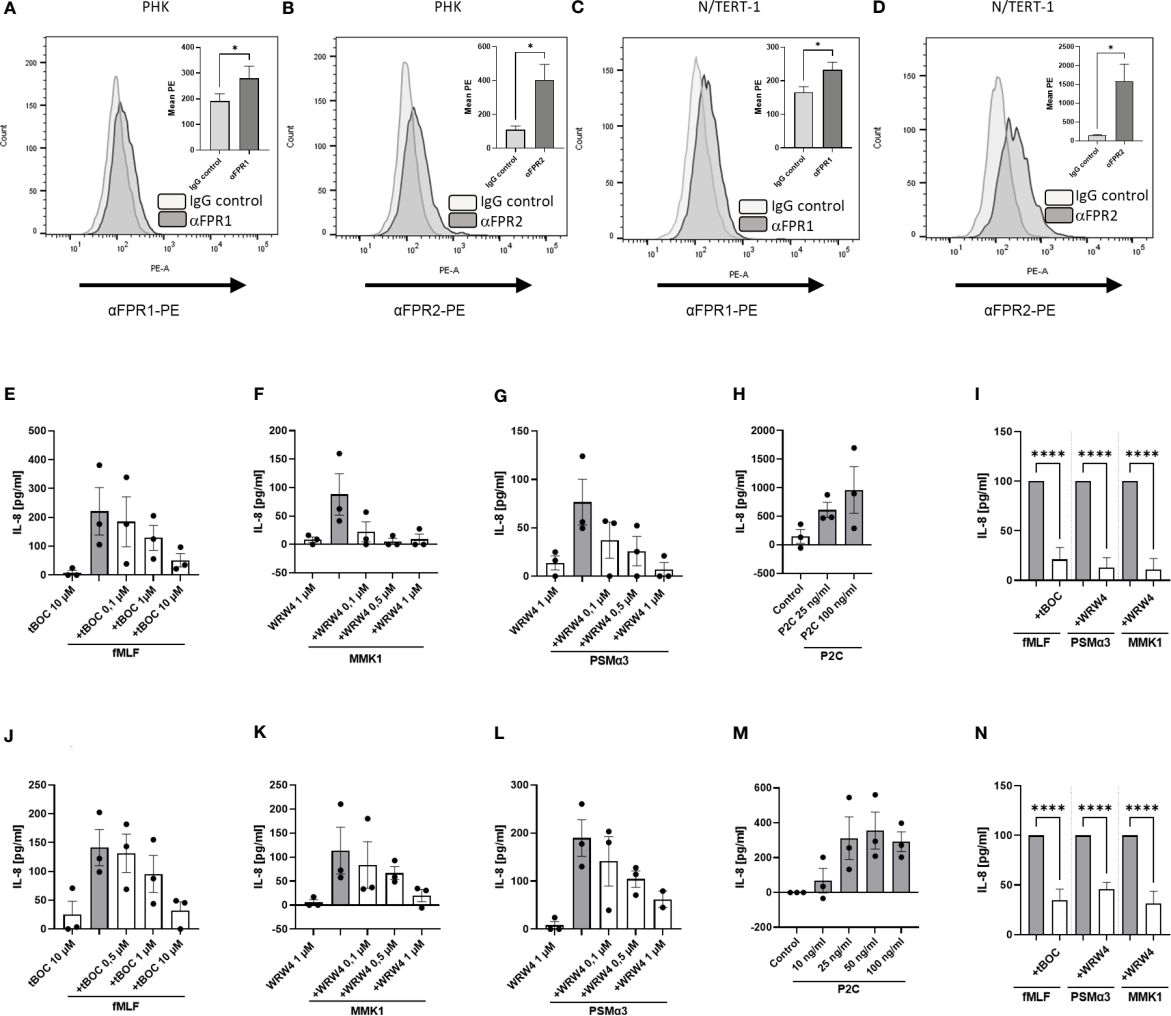
FPR expression and activation induce IL-8 release from keratinocytes in a receptor-dependent manner. FACS analysis of FPR1 and FPR2 expression by PHK **(A, B)** as well as by N/TERT-1 keratinocytes **(C, D)**. IL-8 release of differentiated primary PHKs stimulated for 17 h with either fMLF (100 nM) +/- various concentrations of tBOC **(E)**, MMK1 (100 nM) or PSMα3 (10 nM) +/- various concentrations of WRW4 **(F, G)** or control (DMSO) and P2C **(H)**. Relative amount of released IL-8 by FPR inhibitor-treated keratinocytes (WRW4 1µM, tBOC 10µM) compared to indicated FPR ligands alone **(I)**. IL-8 release of differentiated N/TERT-1 keratinocytes stimulated for 17 h with either fMLF (100 nM) +/- tBOC **(J)**, MMK1 (100 nM) +/- WRW4 **(K)**, PSMα3 (10nM) +/- WRW4 **(L)** or control (DMSO) and P2C **(M)**. Relative amount of released IL-8 by FPR inhibitor-treated N/TERT-1 keratinocytes (WRW4 1µM, tBOC 10µM) compared to indicated FPR ligands alone **(N)**. Data represent mean and SEM out of five experiments **(A, B)**, mean and SEM of six experiments **(C, D)**, of at least 3 experiments (**E–H**, **J–M**), of 4-6 experiments **(I, N)**. All data in **(E–N)** represent IL-8 release minus IL-8 release by medium treated cells. *P < 0.05; ****P < 0.0001, significant difference versus the indicated FPR ligands as calculated by paired two-tailed Student’s t-tests of the mean **(A–D)** or ordinary one-way ANOVA of baseline corrected data **(I, N)**.

It has been described that keratinocytes express FPRs (11). To analyze consequences of FPR activation in keratinocytes, we evaluated the suitability of the immortalized human keratinocyte cell line N/TERT-1 for such functional studies since primary human keratinocytes (PHK) have only a limited lifespan. We compared the expression of FPRs on the surface of PHK (**Figures 1A, B**) with the expression by differentiated N/TERT-1 cells (**Figures 1C, D**). Fluorescence-activated cell sorting (FACS) analysis confirmed FPR expression on both cell types. In order to elucidate whether FPR ligands can also activate keratinocytes, we stimulated PHK and N/TERT-1 with synthetic FPR1 and FPR2 ligands and included the TLR2 ligand Pam2Cys (P2C) as a positive control. It is well documented that staphylococci release TLR2 ligands (20) and TLR2 activation in keratinocytes leads to IL-8 release (21). We assessed whether FPR activation by the FPR1 ligand fMLF, the staphylococcal FPR2 ligand phenol-soluble modulin α3 (PSMα3), or the synthetic FPR2 ligand MMK1 can trigger the release of proinflammatory cytokines by differentiated PHK. We observed that stimulation of PHK with FPR1 or FPR2 ligands increased the release of IL-8 (**Figures 1E–G**). However, activation of TLR2 by the lipopeptide P2C increased the release of these cytokines even stronger (**Figure 1H**). To confirm that the observed effects are FPR-dependent, we incubated the cells with increasing concentrations of the FPR1- or FPR2-specific inhibitors tBOC or WRW4, respectively, to prevent PHK activation. We observed dose-dependent, significant inhibition of the FPR ligand-induced release of IL-8 (**Figure 1I**). Moreover, stimulation of differentiated N/TERT-1 cells led to comparable results with respect to IL-8 release (**Figures 1J–N**). We concluded that these cells can be used as surrogate for PHK and analyzed **S. epidermidis*-*derived FPR2 ligands PSMα and PSMε regarding IL-8 induction in N/TERT-1. Again, IL-8 release was inhibited by WRW4 (**Supplementary Figure 1A**).

### 2.2 Activation of FPRs induces expression of IL-1α, CXCL10, and MIP-3α in keratinocytes

**Figure 2:**
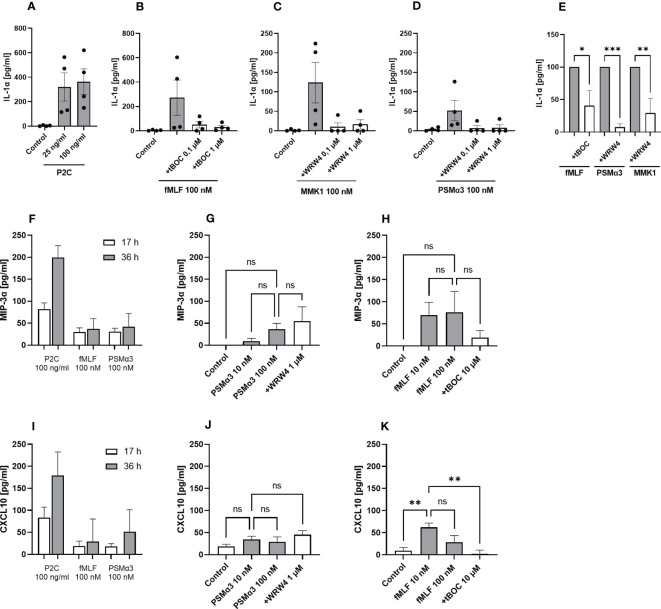
Activation by FPR ligands induces IL-1α as well as MIP-3α and CXCL10 release from keratinocytes. IL-1α release of differentiated N/TERT-1 keratinocytes stimulated for 17 h with either P2C **(A)**, fMLF +/- tBOC **(B)**, MMK1 +/- WRW4 **(C)**, or PSMα3 +/- WRW4 **(D)** and control (DMSO). Relative amount of released IL-1α by keratinocytes treated with FPR inhibitors as compared to indicated FPR ligands alone **(E)**. MIP-3α release of differentiated N/TERT-1 keratinocytes stimulated for 17 or 36 h with either P2C, fMLF or PSMα3 **(F)**. MIP-3α release after 36 h stimulation with different concentrations of PSMα3 +/- WRW4 **(G)** or fMLF +/- tBOC **(H)** and control (DMSO). CXCL10 release of differentiated N/TERT-1 keratinocytes stimulated for 17 or 36 h with either P2C, fMLF or PSMα3 **(I)**. CXCL10 release after 36 h stimulation with different concentrations of PSMα3 +/- WRW4 **(J)** or fMLF +/- tBOC **(K)** and control (DMSO). Data in A-K represent means ± SEMs from at least four independent experiments. Data in **(A–H)** represent cytokine release minus cytokine release by medium-stimulated cells. Ns, not significant, *P < 0.05; **P < 0.01; ***P < 0.001, significant difference versus the indicated FPR ligands as calculated by ordinary one-way ANOVA of baseline corrected data **(E)** or one-way ANOVA with Dunnett’s multiple comparisons test **(G, H, J, K)**.

Next, we were interested whether keratinocyte FPR activation leads to secretion of other chemokines or cytokines in addition to IL-8. Therefore, we analyzed N/TERT-1 cells for the release of IL-1α, CXCL11, CCL2, CCL3, MIP-3α and CXCL10 (**Figure 2**; **Supplementary Figure 2**). Besides the TLR2 ligand P2C, the FPR1 ligand fMLF, the *S. aureus* FPR2 ligand PSMα3, the *S. epidermidis* FPR2 ligands PSMα and PSMε as well as the synthetic FPR2 ligand MMK1 induced IL-1α release from keratinocytes (**Figures 2A–D**; **Supplementary Figure 1B**). Significant inhibition of FPR ligand-induced release of IL-1α was achieved with 1 µM WRWR4 or 10 µM tBOC (**Figure 2E**; **Supplementary Figure 1B**), thereby confirming that the induction was mediated by FPR2 and FPR1, respectively. fMLF and PSMα3 also induced MIP3-α and CXCL10 slightly, which did not markedly increase after prolonged stimulation (**Figures 2F, I**). Whereas induction of these two chemokines by fMLF could be inhibited by tBOC, and reached statistical significance at least for CXCL10 (**Figures 2H, K**), induction by PSMα3 was not inhibited by WRW4 suggesting that induction of CXCL10 and MIP3-α by PSMα3 is not FPR2-dependent and may be a secondary consequence of the membrane-perturbing properties of PSMα3 (**Figures 2G, J**).

### 2.3 Influence of FPRs on keratinocyte proliferation, migration, and gap closure

**Figure 3:**
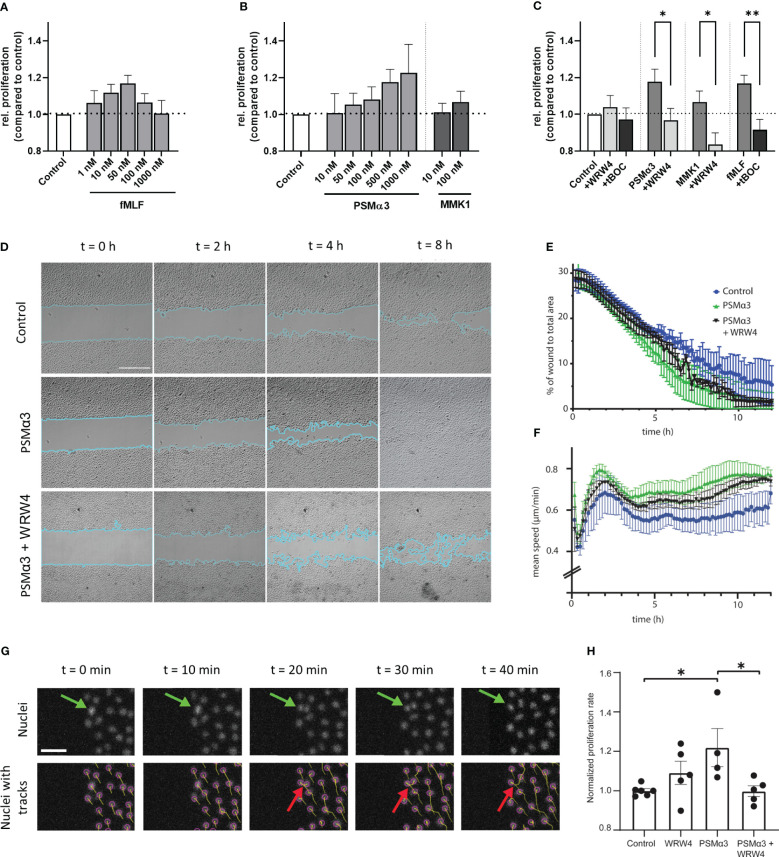
Influence of FPR ligands and inhibitors on proliferation, wound closure efficiency and migration speed. Relative proliferation of 24 h stimulated PHKs either treated with different concentrations of fMLF **(A)** or different concentrations of FPR2 ligands PSMα3 and MMK1 **(B)** compared to untreated (medium) control. Influence of FPR1 inhibitor tBOC (10 µM) on fMLF (50 nM) enhanced proliferation or FPR2 inhibitor WRW4 (1 µM) on PSMα3 (500 nM) or MMK1 (100 nM) induced proliferation **(C)**. Representative phase contrast images of wound closure of undifferentiated N/TERT-1 keratinocytes treated with vehicle control (top row), 10 nM PSMα3 (middle row) or 10 nM PSMα3 plus 1 µM WRW4 (bottom row). Scale bar: 500 µm. Cyan line traces the wound edges **(D)**. Percentage of gap (i.e., wound) area compared to the area of the whole field of view (y-axis) as a function of time (x-axis, h) **(E)**. Mean migration speed (y-axis, μm/h) of N/TERT-1 cells as a function of time (x-axis, h) for cells repopulating the wound as in previous panels **(F)**. Exemplary epifluorescence images of N/TERT-1 keratinocytes’ nuclei stained with live-cell DNA dye (Hoechst) during wound closure. Top row shows nuclei without tracks, while bottom row shows the same images with superimposed object detection (purple circles) and tracks (cumulative displacements of the nuclei). Green arrows mark a dividing cell (on top) and cell division as detected by the nuclear segmentation and tracking (bottom row, red arrows) **(G)**. Using Trackmate Fiji plugin, cells were tracked for 12 h, split events were counted and divided by the total number of nuclei. Cells were treated with PSMα3 (10 nM), WRW4 (1 µM), both or vehicle control. Proliferation rate was normalized with respect to the mean of cells treated with vehicle control **(H)**. Data represent mean and SEM of six independent experiments of three different donors, **(A-C)** or three independent experiments **(D-H)**. *P < 0.05; **P < 0.01, significant difference versus the indicated FPR ligands as calculated by paired two-tailed Student’s t-test **(C)**. A one-way ANOVA followed by a Dunnett’s multiple comparison test was performed and control (*P = 0.02) and PSMα3 plus WRW4 treated cells’ proliferation rate (*P = 0.02) was determined to be significantly altered compared to PSMα3 treated cells only **(H)**.

A typical feature of skin lesions in atopic dermatitis is abnormally enhanced keratinocyte proliferation (22, 23), which may be shaped by skin microbiome members via FPR stimulation. To assess this possibility, we analyzed whether proliferation of non-differentiated PHK may be influenced by FPR activation or inhibition using a proliferation assay, based on the tetrazolium salt WST-1, which can be metabolized by active cells to formazan. We observed that low concentrations of FPR ligands promote the proliferation of PHK in a dose-dependent trend (**Figures 3A, B**), whereas inhibitors of FPR1 and FPR2 prevented the proliferation-stimulating activity of FPR ligands (**Figure 3C**). To assess whether enhanced proliferation may support the skin wound healing process, we monitored wound gap closure in a scratch assay with cells seeded on coverslips. Live-cell imaging of cells populating the wound was performed for 12 hours. Since cultivation of PHK is limited to very few passages, we used N/TERT-1 keratinocytes instead of PHK. We analyzed how fast cells populate the wound either with addition of FPR2 ligand PSMα3 alone or in combination with the FPR2 inhibitor WRW4. To stain the nucleus of living cells, Hoechst was used, which allowed us to segment cell nuclei and follow their tracks during wound healing as well as to determine nuclei splitting events by performing image processing on the microscopy image data (**Figure 3G**). Significantly improved gap closure, particularly in the case of PSMα3-treated cells compared to medium control, was observed, whereas inhibition of FPR2 by WRW4 delayed PSMα3-mediated enhanced gap closure (**Figures 3D, E**; **Supplementary Movie 1**). Furthermore, the mean cell migration speed was significantly increased in cells treated with PSMα3 compared to the medium control, and interestingly, even after closing the gap, when the cells mix to rearrange the cell monolayer, the migration speed of these cells was still higher (**Figure 3F**, after 7h). Using an orthogonal approach based on tracking cell division events using the imaging data (**Figure 3G**), we also confirmed enhanced splitting events, *i.e.*, proliferation, only for PSMα3-treated cells as compared to medium control, but not for WRW4-treated or for PSMα3 and WRW4-treated cells (**Figure 3H**). Thus, we can conclude that PSMα3-treated cells, which exhibit the greatest wound healing efficiency, achieve that by means of both enhanced proliferation and migration.

### 2.4 FPR2 inhibition prevents inflammation of the skin and enhances colonization by *S. aureus*

**Figure 4:**
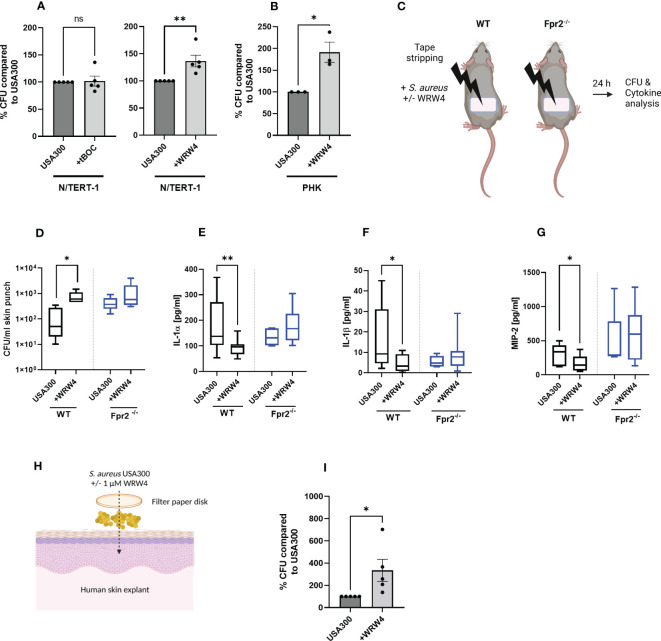
FPR2 inhibition prevents inflammation of the skin and enhances colonization by *S. aureus*. CFUs of *S. aureus* recovered from infected N/TERT-1 **(A)** treated either with USA300 +/- tBOC (10 µM) (left) or USA300 +/- WRW4 (1 µM) (right), or CFU of *S. aureus* recovered from infected PHK +/- WRW4 (1 µM) **(B)**. Skin of tape stripped wild-type and Fpr2^-/-^ were colonized for 24 h with *S. aureus* either with addition of WRW4 or PBS **(C)**. Recovered *S. aureus* **(D)** and IL-1α **(E)**, IL-1β **(F)** or MIP-2 **(G)** derived from supernatants of skin punches of *S. aureus* colonized skin. Model of colonization of human skin explants with *S. aureus* **(H)**. Bacteria derived from human skin explants after *S. aureus* colonization in combination with DMSO or WRW4 (1 µM), **(H, I)**. Data represent mean and SEM of five independent experiments of baseline-corrected data **(A)**, of three independent experiments of three different donors **(B)** of three/four mice per group (WT) and three mice per group Fpr2^-/-^ **(D)** six mice per group (WT) and three mice per group of Fpr2^-/-^ **(E–G)**, two tapes per mouse and of five independent experiments with skin explants from 5 donors **(I)**. *P < 0.05; **P < 0.01, significant difference versus the indicated controls as calculated by paired or two-tailed Student’s t-tests **(A, B, G, I)** or Mann-Whitney test **(D, E)**.

*S. aureus* may reach live keratinocytes in deeper skin areas if the stratum corneum is breached. To analyze if FPR stimulation may affect the capacity of keratinocytes to bind *S. aureus*, we exposed human keratinocytes to *S. aureus* USA300, which produces FPR-activating formylated peptides and PSM peptides, with and without addition of the FPR1 inhibitor tBOC or FPR2 inhibitor WRW4 (**Figure 4A**). Inhibition of FPR2 in PHK as well as in N/TERT-1 significantly enhanced binding by USA300, but not by *S. epidermidis*, whereas inhibition of FPR1 had no influence on *S. aureus* binding (**Figures 4A, B**; **Supplementary Figure 3**). Thus, sensing of PSMs by FPR2 may reduce the capacity of *S. aureus* to bind to keratinocytes, which may help to limit the persistence of *S. aureus* in atopic skin.

Since *S. aureus* skin colonization and subsequent skin inflammation plays a crucial role in atopic dermatitis, we analyzed the influence of FPR2 on *S. aureus* colonization in an *in vivo* model that simulates human atopic dermatitis. We impaired the skin barrier by tape stripping in wild-type and Fpr2^-/-^ mice and applied then PSMs-producing *S. aureus* USA300 with or without WRW4 to the tape-stripped skin (**Figure 4C**). After 24 hours, Fpr2^-/-^ mice contained approximately tenfold higher bacterial numbers on their skin than wild-type mice, indicating that FPR2 plays a critical role in limiting *S. aureus* skin colonization. In support of this finding, WRW4 treatment also increased the abundance of *S. aureus* on the skin of wild-type mice, but it had no influence on the *S. aureus* growth rate on Fpr2^-/-^ skin (**Figure 4D**). These increases were accompanied by a reduction in the secretion of MIP-2, IL-1α, and IL-1β (**Figures 4E–G**) in the skin of Fpr2^-/-^ mice or WRW4-treated wild-type mice compared to untreated wild-type mice. In contrast to the wild-type mice, we observed no difference between WRW4 and mock treatment in the Fpr2^-/-^ mice with respect to release of MIP-2, IL-1α, and IL-1β (**Figures 4E–G**). Interestingly, also inhibition of FPR2 in human skin explants enhanced *S. aureus* binding and colonization, respectively (**Figures 4H, I**).

## 3 Discussion

Secreted bacterial molecules can influence skin homeostasis. Lipopeptides, but also formylated peptides such as PSMs, belong to those molecules that are released by skin colonizing bacteria (19, 20). FPR2 is one of the most promiscuous receptors within the class-A subfamily of GPCRs, not only being able to interact with a variety of ligands but also being expressed by highly diverse cell types (24). In contrast to neutrophils, it was rather unclear if and how FPR2 ligands such as staphylococcal PSMs can influence the physiology of keratinocytes.

It has been described that bacterial colonization promotes wound healing of the skin. Germ-free mice show impaired wound healing compared to specific pathogen-free as well as wild-type mice and treatment of human skin with antibiotics impaired wound healing in an IL-1 receptor-dependent manner (25). Furthermore, FPR ligands were shown to promote cutaneous wound healing (13). Our finding that bacterial FPR ligands promote keratinocyte proliferation in a FPR-dependent manner is in line with these observations (11). Therefore, we speculate that bacterial FPR ligands are responsible for the wound healing-promoting properties of skin bacteria. *S. aureus* skin colonization in patients with AD activates keratinocytes (26) leading to enhanced chemokine and IL-1α production, keratinocyte proliferation, as well as antimicrobial activity. Since we show that all these responses can be triggered by FPR activation and since *S. aureus* is known to produce high amounts of PSMs, we suggest that this is most probably driven by FPR2 (19). This is supported by the fact that expression of a functional Agr global regulatory system in *S. aureus* was not only required for PSM production, but also for epidermal colonization and the induction of AD-like inflammation in mice (27). The enhanced chemokine production could lead to recruitment of leukocytes, which may then promote the elimination of bacteria from infected wounds (28).

However, since the skin barrier of AD patients is frequently impaired, constant FPR activation could be a reason for the observed hyperproliferation of keratinocytes in AD (23), thereby supporting chronic *S. aureus* colonization and maintaining inflammation.

In summary, our data indicate that *S. aureus* furthers skin colonization by modulating a keratinocyte-induced, pro-inflammatory milieu as assessed by cytokine levels via FPRs. Neutrophils also express FPR2 and play a crucial role during skin infection (28). Therefore, in our *in vivo* model it cannot be excluded that cytokine release could be due to leukocyte-derived cytokines, but our *in vitro* data with primary keratinocytes and skin explants suggest that this is rather unlikely. *S. aureus*-triggered chemokine production is accompanied by significant secretion of IL-1α and IL-1β in the skin of wild-type mice after *S. aureus* colonization as observed by us and others (25). It has been described that inhibition of FPRs prevents inflammation of the skin (29). Our data support these observations since inhibition of FPR2 by WRW4 or lack of Fpr2 prevented the release of IL-1α, IL-1β and MIP-2. However, the reduced inflammation led to enhanced skin colonization of *S. aureus.* Hence, it is tempting to speculate that by secreting inhibitors of FPRs, *S. aureus* tries to reduce inflammation. Indeed, *S. aureus* frequently releases inhibitors of FPRs, namely, the proteins CHIPS (30), FLIPr (31) or FLIPr-like (32). Since these inhibitors are human specific, only the *S. aureus*-released agonists of FPRs, i.e., the PSMs, but not the antagonists can be analyzed in animal models. Our data regarding application of the synthetic FPR2 inhibitor WRW4 to the skin of mice, human keratinocytes, or skin explants support the hypothesis that *S. aureus* could use FPR inhibitors to promote skin colonization. The production of FPR ligands and inhibitors may contribute to the ability of *S. aureus* to adjust a favorable balance between activation and inhibition. Our data regarding *S. epidermidis* show that their PSMs also activate keratinocytes in a FPR2-dependent manner. Since colonization of keratinocytes by *S. epidermidis* could not be influenced by the addition of WRW4, the amount of PSMs released by these bacteria might be too low for FPR2 activation. This is consistent with the fact that coagulase-negative staphylococci generally release lower amounts of FPR2 ligands compared to highly pathogenic *S. aureus* strains (9).

It has been shown that mice spontaneously developing AD symptoms due to the lack of cathepsin E exhibit increased susceptibility to infection with *S. aureus* and *Porphyromonas gingivalis* (33). In AD patients with secondary *S. aureus* infections, combination therapy with anti-staphylococcal medicines and topical immunosuppressive corticosteroids improves clinical outcomes more than therapy using topical corticosteroids alone, supporting the significance of a co-occurring *S. aureus* infection in exacerbating the symptomatology of AD patients. Interestingly, FPR-encoding genes belong to the most important hub genes involved in the pathogenesis of acne (34), thus providing further evidence for a role of FPR in the maintenance of inflammatory skin conditions. Our data imply that FPR2 inhibition reduces local activation and cytokine secretion of keratinocytes via FPR2, allowing *S. aureus* to initially colonize better. This increased colonization could then result in the permanent recruitment of more leukocytes, which tend to promote inflammation and *S. aureus* colonization (6).

Based on our findings, topical FPR2 inhibition could be useful as a therapeutic option to reduce sterile inflammation of the skin as described (14). On the contrary, in patients with atopic dermatitis, inhibition of FPR2 in keratinocytes promotes chronic *S. aureus* colonization.

## 4 Material and methods

### 4.1 Bacterial strains, culture conditions and peptides

For this study, *S. aureus* USA300 LAC and *S. epidermidis* 1457 were used and cultured in tryptic soy broth (TSB) at 37°C for 17 h over-night under agitation at 160 rpm. All assays were performed with logarithmically growing bacteria. Various agonists and antagonists were used for this study. As FPR2 agonists PSMα3, PSMα, PSMε (35) and MMK1 were used and WRW4 as FPR2 antagonist. Formylated PSMα3 was kindly provided by Stefan Stevanovic (Dep. of Immunology, Tuebingen). MMK1 (LESIFRSLLFRVM), PSMα, PSMε and WRW4 were purchased (EMC microcollections). fMLF (SIGMA Life Science) was used as FPR1 agonist and N-tert-butoxycarbonyl-methionyl-leucyl-phenylalanine (tBOC; Bachem) as antagonist. For stimulation of TLR2, Pam_2_Cys-SKKKK (P2C, Invivogen) was used.

### 4.2 Cell culture

Primary human keratinocytes (PHK) were isolated from juvenile human foreskin after routine circumcision from the Loretto Clinic in Tübingen, as previously described (7, 36). Primary human keratinocytes were cultured in collagen-coated tissue flasks in CnT-BM.1 Basal Medium 1 with supplements (CELLnTEC) at 37°C and 5% CO_2_. Cell passage 2-4 were used for stimulation and adherence experiments. PHK isolation from human foreskin were approved by the ethics committee of the Medical Faculty of the University Tübingen (750/2018BO2). PHK were differentiated by stimulation for 17 h with 1.7 mM CaCl_2_ in keratinocyte base medium without additional supplements (CELLnTEC). Cytokine release by the medium treated cells was subtracted from all values unless otherwise stated.

The immortalized human keratinocyte cell line N/TERT-1 was kindly provided by J. G. Rheinwald (37). N/TERT-1 cells were cultured in cell culture flasks (Greiner Bio-One GmbH, CELLSTAR® TC) in CnT-BM.1 Basal Medium 1 with supplements (CELLnTEC) at 37°C, 5% CO_2_. Forty-eight hours prior to experiments, as well as during experiments, N/TERT-1 cells were differentiated with 1.7 mM CaCl_2_ in epidermal keratinocyte base medium (CELLnTEC).

### 4.3 Stimulation of keratinocytes with agonists and antagonists for GPCRs

Before stimulation, 250 µl/well of N/TERT-1 cells with a concentration of 0,02 × 10^6^ cells/ml were seeded in epidermal keratinocyte medium containing supplements (CELLnTEC) into 48-well plates coated with 25 µg/ml rat tail collagen I (Corning). After incubation for 72 h at 37°C, 5% CO_2_, cells were differentiated for 48 h with 1.7 mM CaCl_2_ in epidermal keratinocyte base medium (CELLnTEC). PHKs from passage three were seeded into collagen coated 48-well plates in a concentration of 0.04 × 10^6^ cells/ml and incubated 72 h in epidermal keratinocyte medium containing supplements (CELLnTEC). No further differentiation prior to experiments was performed for PHKs. Agonist and antagonists were applied to the PHKs and N/TERT-1 cells in fresh epidermal keratinocyte base medium (CELLnTEC) with 1.7 mM CaCl_2_. Agonist and their respective antagonists were applied individually as well as in combination. Cells were stimulated at 37°C, 5% CO_2_ for 17 h or 36 h. After incubation, plates were centrifuged at 200 × g for 5 min. Supernatant was transferred to polypropylene freezing plates (Thermo Scientific) and stored at -80°C until further analysis via ELISA or Bio-Plex® multiplex assay.

### 4.4 Enzyme-linked immunosorbent assay and multiplex cytokine analysis

Typical cytokines and chemokines released by keratinocytes during inflammation and wound healing were analyzed (38–40). For the detection of IL-8 and IL-1α in cell culture supernatant, ELISAs (R&D Systems) were performed according to the manufacturer’s instruction. For the detection of further chemokines in cell culture supernatants, a customized panel for human chemokines was used (GM-CSF, CCL1, CXCL11, CXCL10, MCP-1, CCL22, CXCL9, MIP-1α, MIP-3α, CCL17) and analyzed according to the manufacturer’s instruction (BioRad, Bio-Plex Pro Human Chemokines Standard, Bio-Plex Pro Human Cytokine Screening Panel Standards). For cell culture samples, 50 µl of a 1:4 dilution was used for analysis. For samples originated from mice, customized panels for mice cytokines and chemokines (IL-1α, IL-1β, IL-10, CXCL10, CXCL11, M-CSF, MCP-1, MIP-1α, MIP-2) were used (BioRad, Bio-Plex Pro Mouse Cytokine 1 and 2 standards). Skin wash samples were used undiluted. All samples were measured with the Bio-Plex™ 200 System (BioRad) and analyzed via the Bio-Plex Manager™ Software (Version 6.2, Build 175 by BioRad).

### 4.5 Analysis of keratinocyte proliferation

For the proliferation assay, PHK were seeded at a concentration of 4 x 10^5^ cells/ml in 100 µl basal medium with supplements (CELLnTEC) into 96-well microtiter flat bottom plates. After 24 h incubation at 37°C and 5% CO_2_, 95% H_2_O, cell supernatants were removed and PHK were stimulated either with FPR1, FPR2, or TLR2 ligands (fMLF, PSMα3, P2C) or inhibitors (WRW4, tBOC) alone or in combination at the indicated concentrations for further 24 h. Triton X100 (1%) was used as negative control and medium as proliferation control. Then WST-1 [2-(4-Iodophenyl)-3-(4-nitrophenyl)-5-(2,4-disulfophenyl)-2H-tetrazolium] Proliferation reagent (10 µl/well) (Merck, Germany) was added and incubated for further three hours at 37°C and 5% CO_2_. WST-1 produces a highly water-soluble formazan upon metabolically active cells, allowing a direct colorimetric measurement of cell viability and proliferation. Then the microtiter plate was shaken at 300 rpm for 1 hour in a microplate reader (Clariostar, BMG Labtech Germany) and absorbance was measured according to manufacturer’s instructions. Proliferation was calculated relative to the medium control.

### 4.6 Scratch assay and video microscopy

Glass bottom 12-well plates (Cellvis P12-1.5H-N) were coated with rat tail collagen I (Thermofisher, A1048301). Culture-inserts 2 well for self-insertion (80209, Ibidi) were glued onto the glass coverslips of each well. 2.8 x 10^5^ N/TERT-1 cells resuspended in 70 μl of complete cell medium were seeded into each well of a culture-insert 2 well µ-Dish (35 mm, Ibidi, 80206). After incubation at 37°C and 5% CO_2_ for 24 h, the cells were stained with Hoechst (1 μg/ml, INVITROGEN CORP, H3570) for 10 min at 37°C, then the culture insert was removed, and cells were washed twice with PBS. Each dish was filled with 2 ml Leibovitz L-15 medium with supplements (CELLnTEC, 11540556, Fisher). Depending on the condition, either FPR2 ligand PSMα3 (10 nM), or FPR2 inhibitor WRW4 (1 µM), or both, was added. Multi-channel time-lapse sequences were acquired of the phase contrast image of cells as well as fluorescence of the Hoechst-stained cell nuclei. Images were taken every 10 min for 12 h using an inverted Nikon Eclipse Ti2 with a Prime 95B camera (Teledyne Photometrics) using a 40X 0.60NA super plan fluor ADM ELWD air objective and the NIS-Elements (RRID : SCR_014329) software package. The microscope was surrounded by a box-type incubator (OKOlab) maintained at 37°C.

### 4.7 Image processing and quantitation of cell speed, scratch area, and cell proliferation rate

For each scratch segment imaged, the area of the scratch (area not covered by cells) was determined at each instance of time using ImageJ (version 1.53t 24, 2022) and in particular a plugin for image analysis of *in vitro* scratch wound assays (41). The Trackmate plugin (42) was used to track the nuclei of the cells using a LAP tracker, which also allowed to count nuclei split events for each given track. Using custom-written MATLAB (MathWorks) scripts, only cells present in the first-time frame were considered, and based on their tracks, we calculated the mean speed of those cells overtime. We also calculated the percentage of this cell sub-population that divided within the 12-h time frame to determine average cell proliferation rates. The codes with example data can be found at https://github.com/ebastoun/N/TERT_1_kinematics. All scripts and functions are written in MATLAB (MathWorks). Averaging across different independent experiments as well as subsequent statistical analysis was performed using the commercial software Prism (GraphPad).

### 4.8 Keratinocyte adhesion and invasion assay

For adhesion assays, PHK were seeded onto 24-well plates 0.4 µm, (Sarstedt) coated with 25 µg/ml collagen I (rat tail, Corning). When cells were 100% confluent, PHKs were differentiated with differentiation medium (1.7 mM CaCl_2_ in CnT-BM.1 basal medium 1 (CELLnTEC) without supplements) for 24 h at 37°C and 5% CO_2_. PHKs were treated with 1 µM WRW4 or DMSO and were incubated with *S. aureus* USA300 in differentiation medium (multiplicity of infection= 30 (MOI30); optical density (OD)= 0.5) for 1.5 hours at 37°C. After two washing steps with 1x PBS, keratinocytes were lysed with saponin (0,5% in PBS), and serial dilutions of the lysate were plated onto blood agar plates. After overnight incubation at 37°C, colony forming units (CFUs) were counted.

2 ml of N/TERT-1 cells with a concentration of 0.04 x 10^6^ cells/ml were seeded into 6-well plates (Greiner bio-one) coated with 25 µg/ml collagen I (rat tail, Corning). When N/TERT-1 cells were confluent, they were differentiated for 48 h with 1.7 mM CaCl_2_. On the day of infection, USA300 or *S. epidermidis* 1457 over-night culture (17 h) was diluted in TSB to OD_600_ 0.1 and incubated (37°C, 160 rpm) to regrow to OD_600 _1. The bacteria were further diluted in keratinocyte basal medium with 1.7 mM CaCl_2_ to a MOI of five. Keratinocytes were infected with USA300 either alone or in combination with 1 µM WRW4 or DMSO (medium) as control for 2 h (37°C, 5% CO_2_). After 2 h, supernatant was removed, and cells were washed two times with PBS and lysed with 0.5% saponin. Serial dilutions of the cell lysate as well as a bacterial control incubated without N/TERT-1 cells were plated on TSA plates using an IUL EDDY Jet 2 spiral plater. Plates were incubated over-night at 37°C and CFUs were counted using the IUL Flash & Go instrument.

### 4.9 FPR expression

For FPR surface expression analysis, 2 x 10^5^ PHK or N/TERT-1 cells were incubated in a 96-well V-bottom plate either with the isotype control (mouse IgG1, Becton Dickinson), anti-FPR1 mouse anti-human FPR1 (BD Bioscience) or anti-FPR2, each 5 µl (mouse anti-human FPRL1, Alvedron Freiburg) for 40 min on ice in PBS, after two washing steps, each time using 250 µl PBS. Cells were stained with a second fluorescently labelled antibody (goat anti mouse IgG-PE, Abcam) for 25 min on ice in the dark. After two washing steps, cells were analyzed by flowcytometry (Fortessa X-20, Becton Dickinson) and FlowJo™ software.

### 4.10 Infection of human skin explants

Human skin explants were prepared from juvenile human foreskin after routine circumcision. Fat and vascular tissue were removed, and the skin was cut into small 1-cm^2^ pieces. 30 min before infection, 1 µM WRW4 diluted in PBS or DMSO (1:45000) in PBS, was topically applied onto human skin explants by using 8-mm filter paper discs (Smart Practice). Human skin explants were then infected with 10^8^ *S. aureus* USA300 for 3 h at 37°C. Using a biopsy punch, 8-mm skin punches from infected skin were prepared and washed twice with 1x PBS. Subsequently, human skin explants were cut into small pieces and scraped, and serial dilutions of the skin lysate were plated onto blood agar plates. After over-night incubation at 37°C, CFUs were counted. Experimental procedures for PHK and skin explants were approved by the local medical ethical committee (reference numbers 750/2018BO2, 054/2017BO2).

### 4.11 *In vivo* skin colonization model

Animal studies were performed with 6-8 week-old female C57BL/6J WT and Fpr2^-/-^ mice. *S. aureus* USA300 was inoculated in TSB and grown for 17 h under aerobic conditions followed by two washing steps with PBS. The mouse skin was shaved before experiments under isoflurane anaesthesia. Tape-stripping was conducted as described bevor (7). Therefore, skin of mice was superficially disrupted by repeated stripping with an adhesive tape (7 times) before *S. aureus* application. To analyze *S. aureus* skin colonization, 15 µl of a bacterial suspension containing 10^8^ *S. aureus* and 5 µl PBS or WRW4 (10 µM) was applied epicutaneously for 24 h on the shaved dorsal skin of mice using filter papers and Finn Chambers (Smart Practice, Barsbuttel, Germany) under isoflurane anaesthesia. After 24 h, the mice were euthanized, and 4 mm skin punches were used for analysis of *S. aureus* CFUs as previously described (6, 7). Washing of the skin punch (vortex for 10 s) in PBS yielded the “wash” fraction used for CFU and cytokine/chemokine Multiplex analysis described above. Experimental procedures involving mice were approved by the local authorities (Regierungspräsidium Tübingen, IMIT 3/18G).

### 4.12 Statistics

All statistical analyses were performed with GraphPad Prism 9.0 (GraphPad Software, La Jolla, USA). The amount of cytokine (**Figures 1I, N**, **Figure 2E**) or number of CFUs (**Figures 4A, B, I**; **Supplementary Figure 3A**) released by keratinocytes stimulated with fMLF, MMK1 or PSMα3 was normalized to 100% (baseline corrected) and the inhibition of cytokine release or CFUs by WRW4 related thereto. An unpaired two-tailed Student’s t-test or Mann-Whitney Test (for the animal experiments) was performed to compare two data groups, while more than two data groups were analyzed by one-way ANOVA with Dunnett’s multiple comparisons test if not otherwise noted.

**Figures 4C**, **4H** were created with BioRender.com.

## Data availability statement

The original contributions presented in the study are included in the article/**Supplementary Material**. Further inquiries can be directed to the corresponding author.

## Ethics statement

The studies involving human participants were reviewed and approved by local medical ethical committee (reference numbers 750/2018BO2, 054/2017BO2). The patients/participants provided their written informed consent to participate in this study. The animal study was reviewed and approved by Regierungspräsidium Tübingen.

## Author contributions

ML, MM and DK designed the experiments. ML, MM, JB, DK and JS performed the experiments. ML, AP, EB and DK edited the manuscript and interpreted the data. All authors contributed to the article and approved the submitted version.

## Supplementary material

Supplementary Figure 1 Activation of N/TERT-1 by *S. epidermidis* PSMα and PSMε induces IL-8 and IL-1α release from keratinocytes in a FPR2-dependent manner. IL-8 **(A)** or IL-1α release **(B)** of differentiated N/TERT-1 keratinocytes stimulated for 17 h with either PSMα (1000 nM) +/- WRW4 (1 µM) or PSMε (500 nM) +/- WRW4 (1 µM). Data represent cytokine release minus cytokine release by medium-treated cells. *P < 0.05; **, P < 0.01 ***, significant difference versus the indicated controls as calculated by unpaired Student’s t-tests.

Supplementary Figure 2 Release of cytokines an chemokines by N/TERT-1. CXCL11, MCP-1, GM-CSF and MIP-1α release of differentiated N/TERT-1 keratinocytes stimulated for 17 h with either P2C (100 ng/ml), fMLF (100 nM) or PSMα3 (100 nM). Release of CCL22, CXCL9 and CCL17 were below the detection level. Data represent means ± SEMs from at least four independent experiments.

Supplementary Figure 3 Inhibition of FPR2 does not affect colonization by *S. epidermidis*. CFUs of *S. epidermidis* 1457 recovered from infected N/TERT-1 treated +/- WRW4 (1 µM). Data represent mean and SEM of five independent experiments of baseline-corrected data. Ns = not significantly different versus the indicated control as calculated by paired Student’s t-tests.

Supplementary movie 1 Influence of FPR ligands on keratinocyte wound closure. Top row: phase contrast video of N/TERT-1 cells over 12 h period (frame rate: 10 min), treated with vehicle control (left), or 10 nM PSMα3 (middle), or 10 nM PSMα3 plus 1 µM WRW4 (right) as they migrate to close a wound. Scale bar: 500 µm. Bottom row: video of fluorescently labelled N/TERT-1 cell nuclei stained with a DNA dye (Hoechst), corresponding to the same cells as in the top row. Superimposed are the displacement tracks of all individual cells color-coded for the total distance travelled by each nucleus (blue = 0 µm, red = 500 µm) within the span of the video (12 h).
