# Supplementary figures and images for: Keratinocytes use FPR2 to detect Staphylococcus aureus and initiate antimicrobial skin defense
Source: Front Immunol. 2023 May 31;14:1188555. doi: 10.3389/fimmu.2023.1188555 (PMC10264695; doi:10.3389/fimmu.2023.1188555)

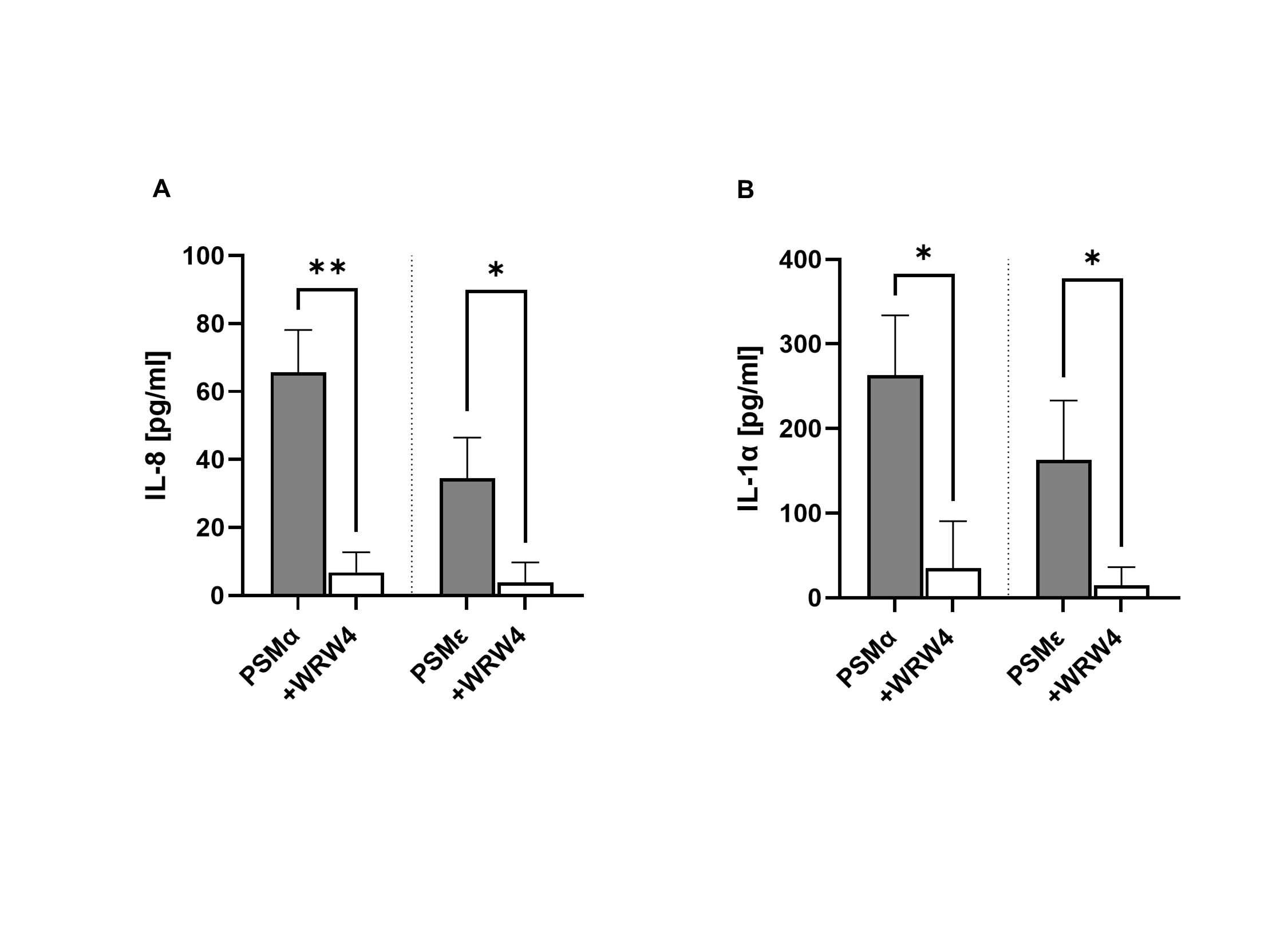

Supplement: Supplementary Figure 1 — Activation of N/TERT-1 by S. epidermidis PSMα and PSMε induces IL-8 and IL-1α release from keratinocytes in a FPR2-dependent manner. IL-8 (A) or IL-1α release (B) of differentiated N/TERT-1 keratinocytes stimulated for 17 h with either PSMα (1000 nM) +/- WRW4 (1 µM) or PSMε (500 nM) +/- WRW4 (1 µM). Data represent cytokine release minus cytokine release by medium-treated cells. *P < 0.05; **, P < 0.01 ***, significant difference versus the indicated controls as calculated by unpaired Student’s t-tests. [file Image_1.tif]

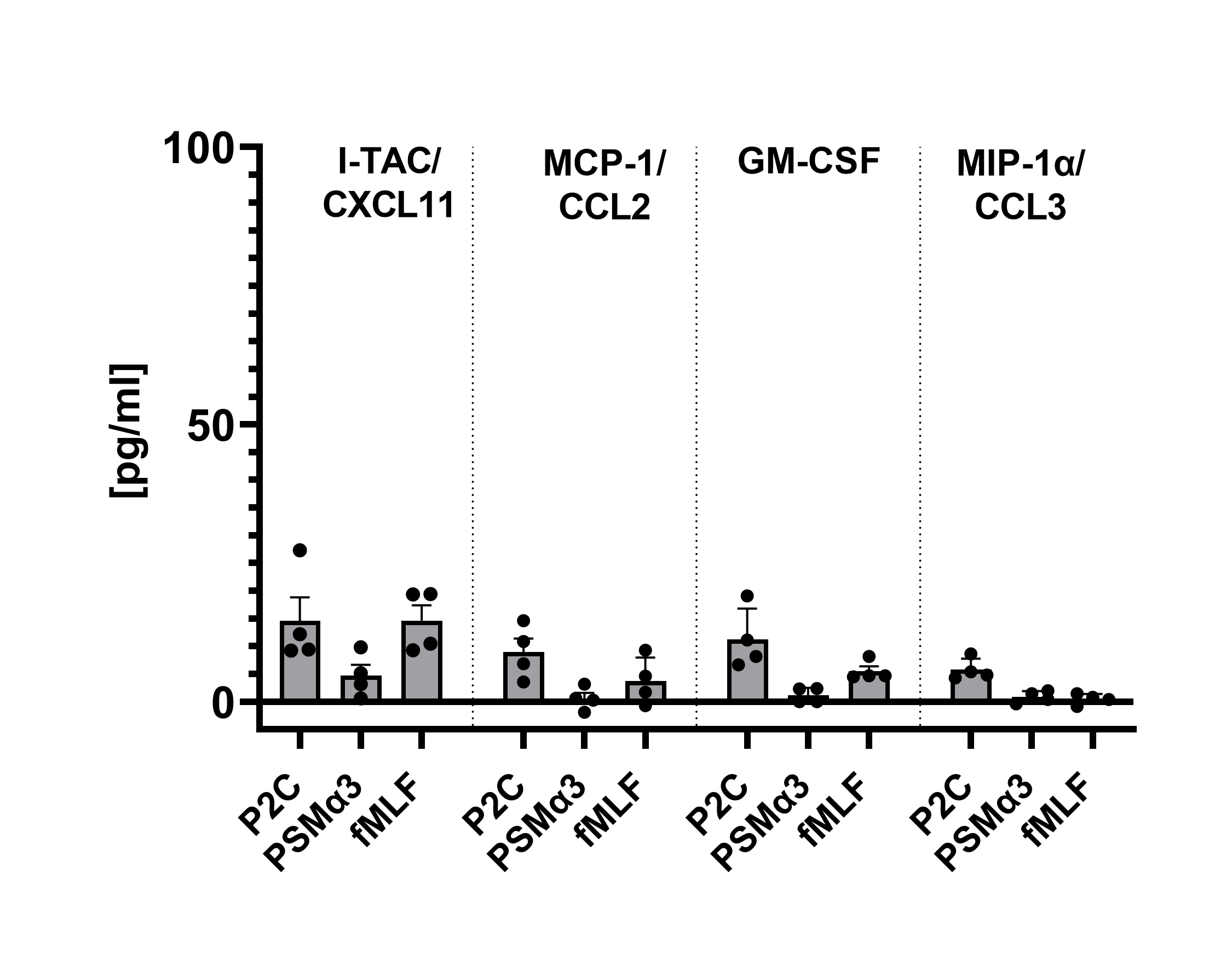

Supplement: Supplementary Figure 2 — Release of cytokines an chemokines by N/TERT-1. CXCL11, MCP-1, GM-CSF and MIP-1α release of differentiated N/TERT-1 keratinocytes stimulated for 17 h with either P2C (100 ng/ml), fMLF (100 nM) or PSMα3 (100 nM). Release of CCL22, CXCL9 and CCL17 were below the detection level. Data represent means ± SEMs from at least four independent experiments. [file Image_2.tif]

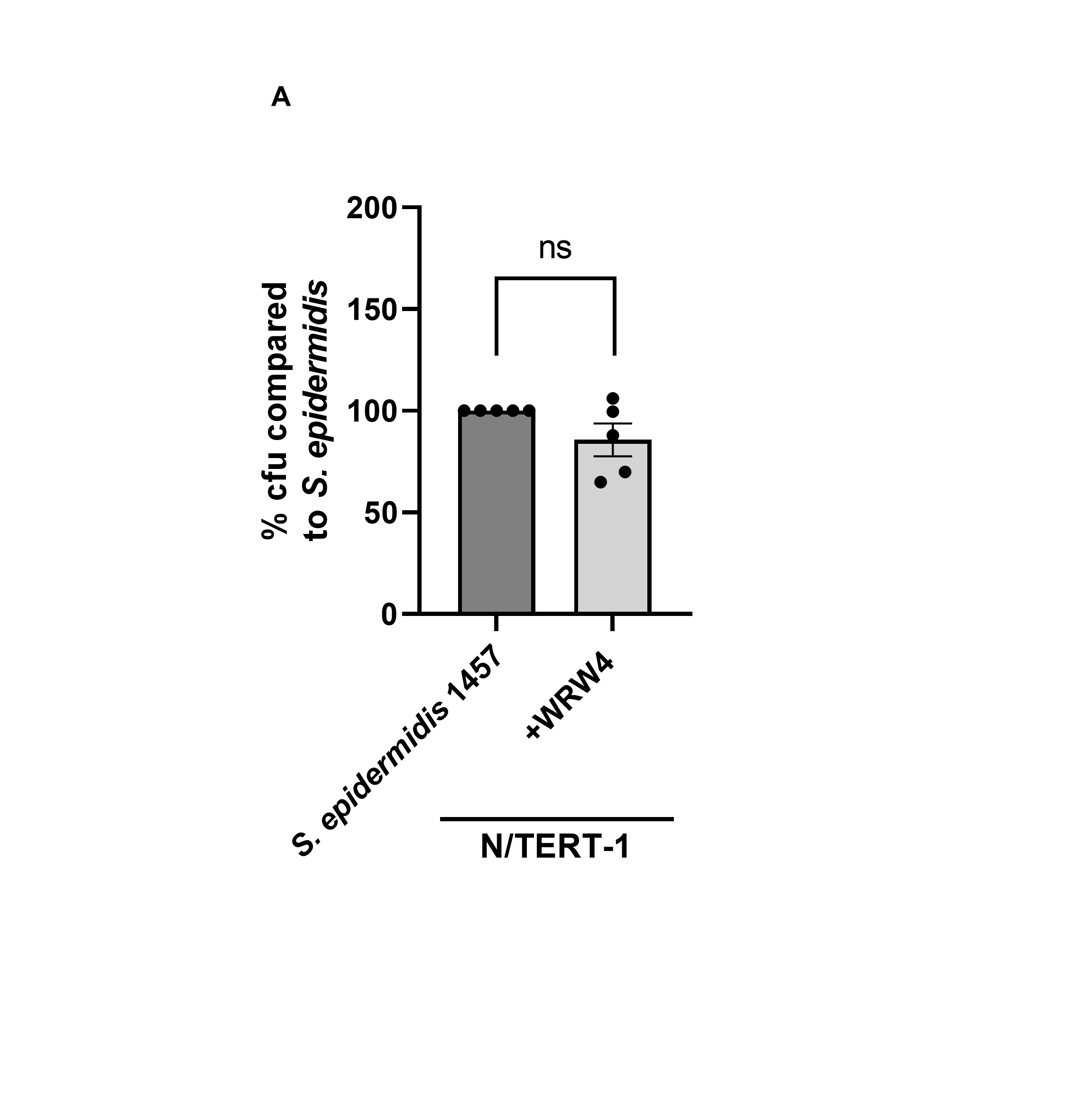

Supplement: Supplementary Figure 3 — Inhibition of FPR2 does not affect colonization by S. epidermidis. CFUs of S. epidermidis 1457 recovered from infected N/TERT-1 treated +/- WRW4 (1 µM). Data represent mean and SEM of five independent experiments of baseline-corrected data. Ns = not significantly different versus the indicated control as calculated by paired Student’s t-tests. [file Image_3.tif]
